# Repurposing the Veterinary Antibiotic Apramycin for Antibacterial and Antibiofilm Activity Against *Pseudomonas aeruginosa* From Cystic Fibrosis Patients

**DOI:** 10.3389/fmicb.2021.801152

**Published:** 2022-02-03

**Authors:** Giovanni Di Bonaventura, Veronica Lupetti, Fabio Verginelli, Sara Giancristofaro, Rosemary Barbieri, Giovanni Gherardi, Arianna Pompilio

**Affiliations:** ^1^Department of Medical, Oral and Biotechnological Sciences, “G. d’Annunzio” University of Chieti-Pescara, Chieti, Italy; ^2^Center for Advanced Studies and Technology (CAST), “G. d’Annunzio” University of Chieti-Pescara, Chieti, Italy; ^3^Department of Pharmacy, “G. d’Annunzio” University of Chieti-Pescara, Chieti, Italy; ^4^Department of Infectious Disease, Istituto Superiore di Sanità, Rome, Italy; ^5^Department of Medicine, Campus Biomedico University, Rome, Italy

**Keywords:** *Pseudomonas aeruginosa*, cystic fibrosis, apramycin, repurposing, biofilm

## Abstract

**Objectives:**

To evaluate the *in vitro* antibacterial, antibiofilm, and antivirulence activities of apramycin, comparatively to tobramycin, against a set of *P. aeruginosa* from chronically infected cystic fibrosis (CF) patients.

**Methods:**

The activity of antibiotics against planktonic cells was assessed by performing MIC, MBC, and time-kill assays. The activity against mature biofilms was evaluated, in a microtiter plate, both in terms of dispersion (crystal violet assay) and residual viability (viable cell count). The effect of drug exposure on selected *P. aeruginosa* virulence genes expression was assessed by real-time Reverse Transcription quantitative PCR (RT-qPCR).

**Results:**

Apramycin MIC_90_ and MBC_90_ values were found at least fourfold lower than those for tobramycin. A comparable trend was observed for mucoid strains. Only 4 out of 24 strains (16.6%) showed an apramycin MIC higher than the epidemiological cut-off value of 64 mg/L, whereas a higher resistance rate was observed for tobramycin (62.5%; *p* < 0.01 vs. apramycin). In time-kill analyses, both aminoglycosides were found bactericidal, although apramycin showed a more rapid effect and did not allow for regrowth. Apramycin generally stimulated biofilm biomass formation, whereas tobramycin showed opposite trends depending on the strain tested. Both drugs caused a highly significant, dose-dependent reduction of biofilm viability, regardless of strain and concentration tested. The exposure to apramycin and tobramycin caused increased expression of *mexA* and *mexC* (multidrug efflux pumps), whereas tobramycin specifically increased the expression of *aprA* (alkaline protease) and *toxA* (exotoxin A). Neither apramycin nor tobramycin showed cytotoxic potential toward IB3-1 bronchial epithelial CF cells.

**Conclusion:**

Our results warrant future pharmacokinetic and pharmacodynamic studies for supporting the rationale to repurpose apramycin, a veterinary aminoglycoside, for CF lung infections.

## Introduction

Cystic fibrosis (CF) is an autosomal disorder caused by a mutation in the CF transmembrane conductance regulator gene (CFTR), leading to an absent or dysfunctional CFTR protein with consequent alteration in the ionic transport (Cutting, 2015; Bergeron and Cantin, 2019). In the lungs, this mutation causes an accumulation of dry and sticky secretions, creating the perfect environment for bacterial infections. Among these infections, those caused by *Pseudomonas aeruginosa* are predominant, especially in adulthood (Maiden et al., 2018). Due to the altered microenvironment of the CF lung, inflammation fails in clearing the infection, thus causing the progression of lung disease that eventually leads to bronchiectasis and death (Chmiel et al., 2002).

Inhaled tobramycin is recommended for the eradication of *P. aeruginosa* infection in CF patients (Mogayzel et al., 2014; Maiden et al., 2018). However, despite intensive antibiotic therapy, the infection is virtually impossible to eradicate because of antibiotic resistance which is further aggravated by the ability of *P. aeruginosa* to grow as biofilm, an aggregation of microorganisms enclosed in an extracellular polymeric substance (EPS) and intrinsically tolerant to antimicrobials and host immune response (Hall-Stoodley et al., 2004; Keren et al., 2004; Ciofu et al., 2015; Maiden et al., 2018). In addition, as an adaptative response to the stressing CF airways, *P. aeruginosa* converts to a mucoid phenotype that is often associated with a poor prognosis for the CF patient, due to an overproduction of alginate and the generation of a thicker extracellular polysaccharide matrix (Folkesson et al., 2012; Maiden et al., 2018). This scenario raises the urgent need to develop new molecules to avoid selecting resistant strains and possibly target cells within sessile communities.

Apramycin is a monosubstituted deoxystreptamine exclusively used in veterinary medicine for the treatment of bovine mastitis and diarrheal disease in farm animals (European Medicines Agency [EMA], 2018). Its chemical structure differs from that of clinically relevant disubstituted aminoglycoside antibiotics, making this molecule intrinsically resilient to almost all resistance determinants typically found in multidrug-resistant (MDR) gram-negative bacteria (Wachino and Arakawa, 2012; Kang et al., 2017). Confirming this, recent studies have shown a broad-spectrum *in vitro* activity of apramycin against human isolates of MDR *Acinetobacter baumannii, P. aeruginosa*, carbapenem-resistant Enterobacteriaceae, and *Staphylococcus aureus* (Truelson et al., 2018; Juhas et al., 2019). In addition, apramycin has been shown to be active against lung and septicemic infections in murine models (Meyer et al., 2014; Becker et al., 2020), with lower toxicity than other disubstituted aminoglycosides (Matt et al., 2012).

Based on these compelling properties, for the first time in the present study the *in vitro* antibacterial, anti-biofilm, and anti-virulence activities of apramycin were evaluated, comparatively to tobramycin, against a selected set of *P. aeruginosa* CF strains. This is a first step in assessing whether apramycin or potential derivatives of apramycin might serve as lead compounds for future therapeutics against *P. aeruginosa* infections in CF patients.

## Materials and Methods

### Bacterial Strains

Twenty-four *P. aeruginosa* strains—isolated from respiratory specimens of chronically infected CF patients—were tested in the present study (Table 1). Strains resulted in being clonally distinct at PFGE analysis (Pompilio et al., 2020). Both mucoid and non-mucoid strains were enrolled since these variants are often co-isolated in CF sputum, indicating they may have selective advantages withstanding into a stressful environment such as CF lung (Clark et al., 2015; Tai et al., 2017; Malhotra et al., 2018). Furthermore, PaPh32 strain was isolated in a CF lung transplant recipient. After identification using MALDI-TOF mass spectrometry, strains were stored at −80°C until use when they were cultured twice on Mueller-Hinton agar (MHA; Oxoid, Milan, Italy) to restore the original phenotypic traits (i.e., mucoid phenotype, antibiotic susceptibility, etc.).

**TABLE 1:** Susceptibility of *P. aeruginosa* CF planktonic cells.

		Apramycin		Tobramycin	
Strain ID	Phenotype	MIC	MBC	MIC	MBC
Pa7		64	256	2 (S)	4
Pa37	Mucoid	8	16	0.25 (S)	0.5
Pa38	Mucoid	128	256	4 (R)	4
Pa39	Mucoid	32	64	256 (R)	256
Pa40	Mucoid	32	64	256 (R)	256
Pa42	Mucoid	16	32	0.5 (S)	0.5
Pa43	Mucoid	8	16	0.5 (S)	0.5
Pa45	Mucoid	16	16	32 (R)	32
Pa46	Mucoid	32	32	256 (R)	256
Pa47	Mucoid	16	32	64 (R)	64
Pa48		32	64	256 (R)	256
Pa49		32	64	1 (S)	2
Pa50		32	64	1,024 (R)	>1,024
Pa51		16	32	256 (R)	256
Pa52		32	64	1 (S)	2
Pa53	Mucoid	16	16	1 (S)	2
Pa54		8	16	32 (R)	64
Pa55		512	512	>1,024 (R)	>1,024
Pa56		16	16	0.5 (S)	1
Pa57		128	128	512 (R)	1,024
PaPh16		64	128	512 (R)	512
PaPh26		128	256	8 (R)	8
PaPh27		8	8	0.5 (S)	0.5
PaPh32	Mucoid	32	64	64 (R)	128

*MIC and MBC values were measured by the broth microdilution method and expressed as mg/L. In brackets, the categorical interpretations of susceptibility (S) and resistance (R) for tobramycin, considering a susceptibility breakpoint of ≤ 2 mg/L (The European Committee on Antimicrobial Susceptibility Testing, 2021).*

### Antimicrobial Susceptibility Testing

Apramycin and tobramycin were obtained as powder with known potency from Merck KGaA (Darmstadt, Germany). Stock solutions were prepared in reagent grade water and stored at −80°C until use. The activity of drugs against planktonic *P. aeruginosa* cells was evaluated by measuring MIC (Minimum Inhibitory Concentration) and MBC (Minimum Bactericidal Concentration) values. MIC values were obtained in cation-adjusted Mueller-Hinton II broth (CAMHB; Becton, Dickinson & Co., Milan, Italy) using the broth microdilution technique and interpreted according to EUCAST guidelines (The European Committee on Antimicrobial Susceptibility Testing, 2021). *Escherichia coli* ATCC25922 and *P. aeruginosa* ATCC27853 were tested in parallel as quality control strains. MBC values were measured according to CLSI guidelines (National Committee for Clinical Laboratory Standards, 1999). Ten microliters of broth culture from wells showing no visible growth at MIC determination were plated onto MHA. The MBC value was defined as the minimum antibiotic concentration able to eradicate 99.9% of the starting inoculum following incubation at 37°C for 24 h.

### Time-Kill Studies

Time-kill assays were performed according to CLSI recommendations (National Committee for Clinical Laboratory Standards, 1999). Briefly, a standardized inoculum (1–2 × 10^6^ CFU/mL) prepared from an overnight growth on Tryptone Soya Agar (TSA; Oxoid, Milan, Italy) was exposed to several concentrations (0.5x, 1x, 2x, 4x, and 8xMIC) of each drug prepared in CAMHB. Control samples were prepared similarly without exposure to drugs. At prefixed times of incubation at 37°C (1, 2, 3, 4, 5, 6, 12, 16, 20, and 24 h), aliquots were removed, and 10-fold serial dilutions were prepared in PBS for colony counting. Results were expressed by plotting Log (CFU/mL) over time, considering 10 CFU/mL as the limit of detection. Antibiotic carry-over effect was not observed. Bactericidal activity was defined as a ≥ 3 Log (CFU/mL) reduction.

### Screening for Biofilm Formation

All strains were screened for their ability to form biofilm using the microtiter plate method. A bacterial suspension—grown overnight in Trypticase Soy broth (TSB; Oxoid) at 37°C and under dynamic conditions (130 rpm)—was adjusted with sterile TSB to an optical density measured at 550 nm (OD_550_) equals to 1.0 (corresponding to 1–4 × 10^8^ CFU/mL) and diluted 1:100 (vol/vol) using sterile TSB. Two hundred microliters of this standardized inoculum were added to each well of a 96-well polystyrene tissue culture plate (Falcon BD; Becton, Dickinson & Co.). Negative controls were prepared similarly without adding the standardized inoculum. After static 24 h-incubation at 37°C, biofilms were washed twice with PBS (pH 7.2) (Merck KGaA), fixed (60°C for 1 h), stained with Hucker-modified crystal violet (5 min, 200 μL/well) (Sonnenwirth, 1980), and air-dried (37°C, 30 min). Crystal violet was extracted using 33% glacial acetic acid (Merck KGaA) (15 min, 200 μL/well). Finally, biofilm biomass was quantified spectrophotometrically, measuring OD_492_ (Sunrise; Tecan, Milan, Italy) (Pompilio et al., 2020). The percentage of biofilm dispersion caused by drug exposure was calculated as follows: (1 – OD_492_ of test/OD_492_ of untreated control) × 100. According to the criteria proposed by Stepanović et al. (2007) a strain was classified for biofilm formation as follows: non-producer (OD ≤ OD_*c*_); weak-producer [OD_*c*_ < OD ≤ (2 × OD_*c*_)]; moderate-producer [(2 × OD_*c*_) < OD ≤ (4 × OD_*c*_)]; or strong-producer (OD > 4 × OD_*c*_). The cut-off value (OD_*c*_) was defined as the mean OD of negative control + 3 × SDs.

### *In vitro* Activity Against Preformed Biofilm

Twenty-four hour old biofilms formed in a 96-well microtiter plate as described in “Screening for biofilm formation” were exposed to each drug tested at the desired concentrations in CAMHB. Following 24 h-exposure at 37°C under static conditions, the effect against mature biofilm was evaluated in terms of dispersion—using crystal violet assay, as described above—and residual viability by viable cell count. In the latter, biofilms were washed twice with sterile PBS to eliminate the drug and not attached cells, then exposed for 5 min to 200 μL trypsin-ethylenediaminetetraacetic acid 0.25% (Merck KGaA). Detached cells were collected by manual scraping and finally underwent viable cell count onto TSA. The percentage of biofilm viability after drug exposure was calculated as follows: [(CFU/well of test)/(CFU/well of untreated control)] × 100.

### Gene Expression Assay

The effect of drug exposure on the transcription levels of *algD*, *toxA*, *lasI*, *aprA*, *mexA*, *mexB*, and *mexC* virulence genes was assessed by real-time Reverse Transcription quantitative PCR (RT-qPCR). Planktonic cells were exposed to each drug at 0.25xMIC for 20 h at 37°C, washed with PBS, and then directly harvested in Qiazol Lysis Reagent (Qiagen; Milan, Italy), a monophasic solution of phenol and guanidine thiocyanate designed to facilitate lysis and inhibit RNases. RNA was extracted following the manufacturer’s protocol adding chloroform for isolation followed by isopropanol and ethanol washes for purification. After DNase I treatment (Merck KGaA), RNA was checked for purity and quantity by NanoDrop-2000 (Thermo Fisher Scientific Italia Inc., Milan, Italy). RNA quality was assessed by running an aliquot of samples on a denaturing agarose gel stained with ethidium bromide. First-strand cDNA was synthesized from 2 μg of RNA using a High-Capacity cDNA reverse transcription kit (Thermo Fisher Scientific Italia Inc.). Next, gene expression was evaluated using 10 ng cDNA by real-time RT-qPCR assay on QuantStudio™ 7 Pro Real-Time PCR System (Applied Biosystems) using the PowerTrack SYBR Green Master Mix (Thermo Fisher Scientific Italia Inc.). Primers were designed using as a reference the genome of *P. aeruginosa* strain NDTH9845 (GeneBank accession number: CP073080.1) (Supplementary Table 1). Considering that the difference between the melting temperature (Tm) and the annealing temperature (Ta) should be ≤ 5°C, all oligonucleotides were designed with a Tm not exceeding 65°C to use a Ta = 60°C for all amplifications. Specificity was assessed both *in silico* with BLAST and by PCR endpoint under the same real-time RT-qPCR conditions. The ΔΔCt method was applied to determine the relative gene expression in exposed vs. unexposed cells after normalizing on the expression of the *proC* housekeeping gene. The modulation of expression levels was shown as fold change on a log_2_ scale.

### Cytotoxicity Evaluation

The direct cytotoxic effect of each drug was assessed toward IB3-1 bronchial epithelial cells (ATCC#CRL-2777) isolated from a pediatric CF patient who harbored the ΔF508/W1282X mutations within the CFTR gene. Cells were grown as monolayer at 37°C in LHC-8 medium (Thermo Fisher Scientific Italia Inc.) supplemented with 5% fetal bovine serum (Thermo Fisher Scientific Italia Inc.) in a 5% CO_2_ atmosphere. After exposing the monolayer to each drug at the desired concentration for 24 h, the cell viability was measured by an MTS tetrazolium-based colorimetric assay (CellTiter 96^®^ AQueous One Solution Cell Proliferation Assay, Promega, Milan, Italy). Briefly, 20 μL of a mixture of MTS [3-(4,5-dimethylthiazol-2-yl)-5-(3-carboxymethoxyphenyl)-2-(4-sulfophenyl)-2H-tetrazolium] and the electron coupling reagent PES (phenazine ethosulfate) were added to each well containing exposed cells. Untreated IB3-1 cells were prepared as the control. After 4 h-incubation at 37°C, the OD_492_ was measured using an ELISA plate reader (Sunrise, Tecan Trading AG, Switzerland).

### Statistical Analysis

Each experiment was carried out at least in triplicate and repeated on two different occasions (*n* ≥ 6). Statistical analysis was performed using GraphPad software (ver. 8.0; GraphPad Inc., CA, United States). Data distribution was assessed using the D’Agostino & Pearson normality test, and then the statistical significance of differences was evaluated using: (i) ordinary one-way ANOVA followed by Dunnett’s multiple comparisons post-test, for datasets normally distributed; (ii) Holm-Sidak’s or Tukey’s multiple comparisons post-test in case datasets did not pass the normality test. Differences between percentages were evaluated using Fisher’s exact test. The significance level was set at *p* < 0.05. Differences between MIC or MBC values were considered as significant for discrepancies ≥ 2 log_2_ concentration steps.

## Results and Discussion

MIC and MBC values for apramycin and tobramycin toward 24 *P. aeruginosa* CF strains were obtained using the broth microdilution technique, and results are shown in Table 1. A high degree of resistance to tobramycin was observed (15 out of 24 strains, 62.5%). Although it was not possible to make a similar categorical assessment for apramycin due to the lack of established clinical EUCAST/CLSI breakpoints, MIC_90_ and MBC_90_ values (i.e., the lowest concentration of an antimicrobial capable to inhibit or kill 90% of bacterial isolates, respectively) were significantly lower, at least 4–8-fold, than tobramycin (128 vs. 1,024 mg/L, and 256 vs. 1,024 mg/L, respectively). Our findings are concordant with a previous study where the activity of apramycin against MDR *A. baumannii* and *P. aeruginosa* clinical isolates was evaluated (Kang et al., 2017).

A similar trend was observed when considering mucoid strains only, with apramycin MIC_90_ and MBC_90_ values, respectively, 8- and 4-fold lower than tobramycin (32 vs. 256 mg/L, and 64 vs. 256 mg/L, respectively). This might be particularly relevant in CF patients where *P. aeruginosa* mucoid conversion is associated with a worse prognosis (Li et al., 2005; Malhotra et al., 2019).

**FIGURE 1:**
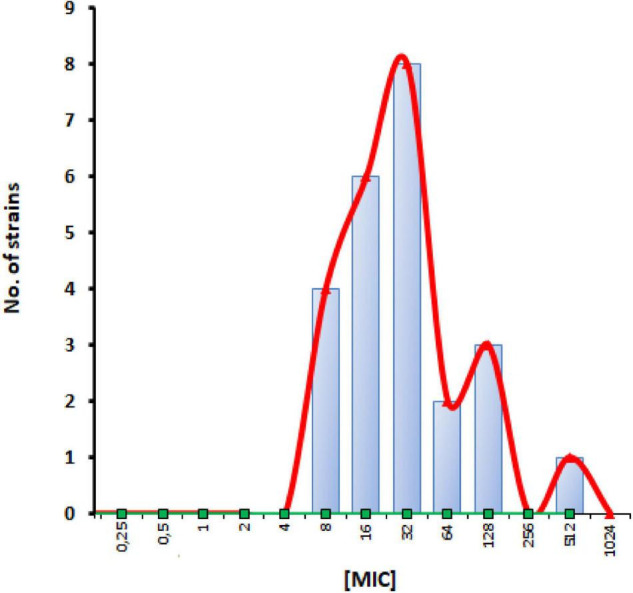
Apramycin MIC distribution for *P. aeruginosa* CF strains. Twenty-four strains were examined in this study. Vertical bar designates an epidemiological cut-off value of 64 mg/L.

**FIGURE 2:**
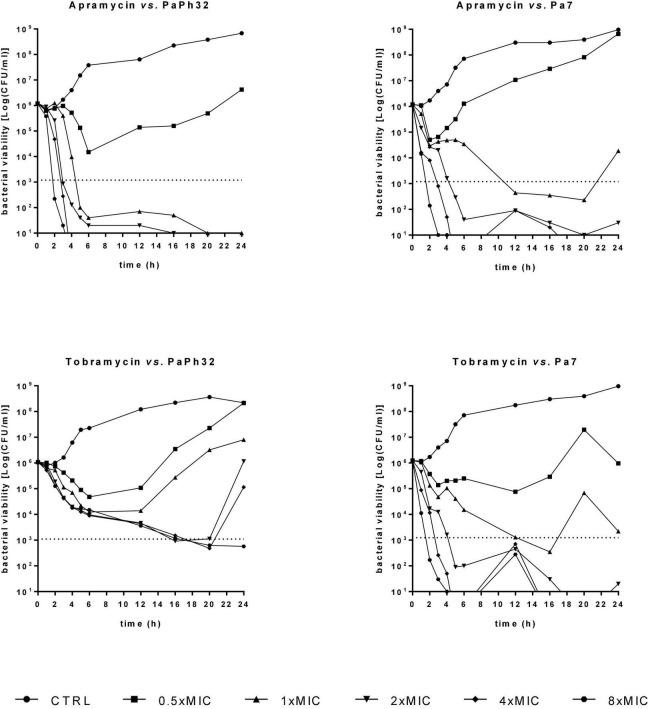
Time-kill kinetics. The kinetics of apramycin and tobramycin activity was carried out over 24 h in liquid medium. *P. aeruginosa* PaPh32 and Pa7 were selected as representative of tobramycin-resistant and -susceptible strains, respectively. Each antibiotic was tested at MIC value (apramycin: 32 and 64 mg/L, respectively, for PaPh32 and Pa7; tobramycin: 64 and 2 mg/L, respectively, for PaPh32 and Pa7), and its fractions and multiples. The dotted line indicates bactericidal activity, defined as a ≥ 3 Log (CFU/mL) reduction of the initial inoculum size. The limit of detection was 10 CFU/ml.

In accordance with previous evidence (Kang et al., 2017), the visual inspection of apramycin MIC distribution suggested an epidemiological cut-off value of 64 mg/L (Figure 1). It is worth noting that only 4 out of 24 (16.6%) strains had an MIC above this cut-off suggesting very low levels of acquired apramycin resistance, significantly lower than that observed for tobramycin (15 out of 24, 62.5%; *p* < 0.01 vs. apramycin). Overall, these findings suggest the intrinsic resilience of apramycin to common mechanisms of aminoglycoside resistance (Davies and O’Connor, 1978; Lovering et al., 1987), in this highly tobramycin-resistant *P. aeruginosa* strain set. Furthermore, no cross-resistance occurred (relationship between MIC values of two drugs; Pearson correlation coefficient r: 0.169). Comparative analysis of MBC and MIC values performed by calculating the killing quotient (KQ = MBC/MIC) indicated that both aminoglycosides are bactericidal (KQ < 4) against all strains tested. The bactericidal effect of apramycin was confirmed by time-to-kill assays performed on PaPh32 and Pa7 strains, respectively, representative for tobramycin-resistant and -susceptible phenotypes (Figure 2). Both aminoglycosides showed dose-dependent bactericidal activity, although to different extents. Indeed, although apramycin and tobramycin showed comparable time-kill kinetic toward Pa7 strain—resulting bactericidal at 8x, 4x, and 2xMIC—apramycin resulted more effective against the tobramycin-resistant PaPh32 strain, proving to be bactericidal already after 2 h-exposure at 8xMIC, and within 6 h-exposure at 4x, 2x, and 1xMIC. Conversely, tobramycin exerted bactericidal effect only at 8x and 4xMIC, although a regrowth was observed at 4xMIC. These findings are consistent with prior observations of rapid bactericidal activity of apramycin toward *N. gonorrhoeae* (Riedel et al., 2019), *A. baumannii* (Kang et al., 2018), and *S. aureus* (Truelson et al., 2018), whereas no data has been published for *P. aeruginosa.* Overall, our data indicate that apramycin might be preferable to tobramycin for rapid, early, time-kill properties against *P. aeruginosa*.

**FIGURE 3:**
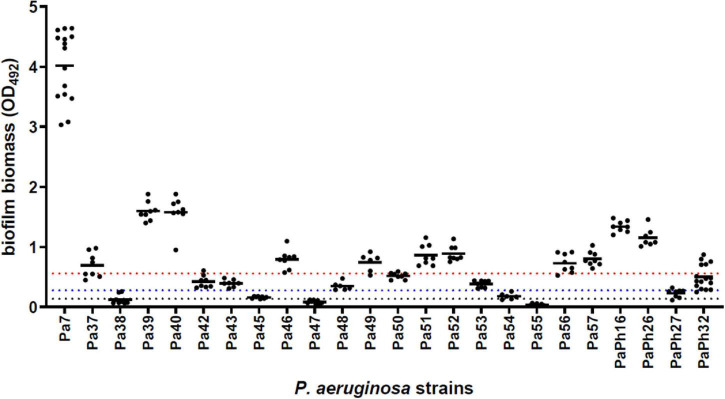
Biofilm formation by *P. aeruginosa* strains from CF patients. The amount of biofilm formed following 24 h-incubation at 37°C was measured in polystyrene 96-well microtiter plate using the crystal violet assay. Results are shown as scatter plot, with the horizontal solid line indicating the mean OD value. According to the criteria proposed by Stepanović et al. (2007) a strain was classified for biofilm formation as follows: non-producer (OD ≤ OD_*c*_; below the dotted black line); weak-producer [OD_*c*_ < OD ≤ (2 × OD_*c*_); between dotted black and blue lines]; moderate-producer [(2 × OD_*c*_) < OD ≤ (4 × OD_*c*_); between dotted blue and red lines]; or strong-producer (OD > 4 × OD_*c*_; above the dotted red line). Cut-off value (OD_*c*_) was defined as the mean OD of negative control + 3 × SDs.

Chronic *P. aeruginosa* CF lung infections evolve to generate environmentally adapted clusters of communities, so-called biofilms, suspended within the airway mucus and inherently resistant to antibiotics and innate host defenses, thus leading to increased morbidity and mortality (Høiby et al., 2010; Alhede et al., 2011). According to the criteria proposed by Stepanović et al. (2007) most strains (21 out of 24, 87.5%) could be classified as biofilm producers, and 50% as strong producers (Figure 3), thus confirming that the biofilm mode of life is relevant to the persistence of *P. aeruginosa* during long-term colonization of CF airways (Rossi et al., 2021).

**FIGURE 4:**
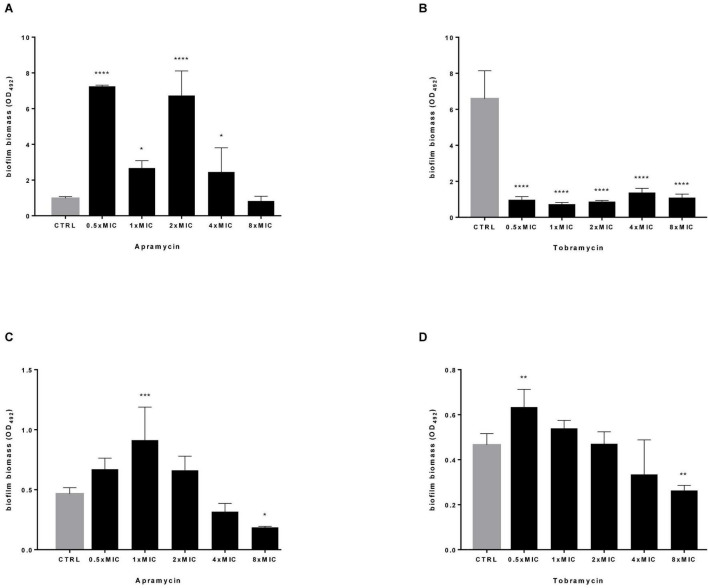
Dispersal activity against preformed *P. aeruginosa* biofilm. The efficacy of apramycin and tobramycin to disperse 24 h-mature biofilm by *P. aeruginosa* Pa7 **(A,B)** and PaPh32 **(C,D)** was assessed using crystal violet assay. Each drug was tested at fractions and multiples of MIC value (apramycin: 64 and 32 mg/L; tobramycin: 2 and 64 mg/L, respectively, for Pa7 and PaPh32). Results are expressed as mean + SD of the residual biofilm biomass (OD_492_) after 24 h-exposure. Control samples (CTRL) were not exposed to drug. Statistical significance at ordinary one-way ANOVA followed by Holm-Sidak’s multiple comparisons post-test: *p < 0.05, **p < 0.01, ***p < 0.001, ****p < 0.0001 vs. CTRL.

In this frame, apramycin and tobramycin were tested for the potential to disperse and kill mature biofilm by *P. aeruginosa*. To this end, Pa7 (tobramycin-susceptible, the strongest biofilm producer, frayed phenotype) and PaPh32 (tobramycin-resistant, moderate biofilm producer, mucoid phenotype) strains were chosen to evaluate the antibiofilm activity dependence on the susceptibility to tobramycin and the amount of biofilm formed. Results from biofilm dispersal assays indicated that the exposure to apramycin generally stimulates the formation of biofilm biomass, consisting of both cells and self-produced EPS, although at different extent depending on strain and concentration considered (Figure 4). Indeed, although lower concentrations seemed to be more effective in stimulating biofilm formation, exposure to apramycin at 8xMIC provoked a significant dispersion of biofilm formed by PaPh32 (OD_492_, mean ± SD: 0.186 ± 0.008 vs. 0.465 ± 0.05, respectively, for exposed and unexposed biofilms; *p* < 0.05). On the contrary, tobramycin caused a significant reduction of biofilm biomass formed by the Pa7 strain regardless of concentration. An opposite trend was observed for PaPh32 strain whose biofilm amount resulted to be significantly increased after exposure to tobramycin at 0.5xMIC (OD_492_, mean ± SD: 0.633 ± 0.079 vs. 0.465 ± 0.05, respectively, for treated and control biofilms; *p* < 0.01), whereas a significant reduction was found after exposure at 8xMIC (OD_492_, mean ± SD: 0.263 ± 0.022 vs. 0.465 ± 0.05, respectively, for treated and control biofilms; *p* < 0.01). Tobramycin did not exert any significant effect when tested at 1x, 2x, and 4xMIC.

**FIGURE 5:**
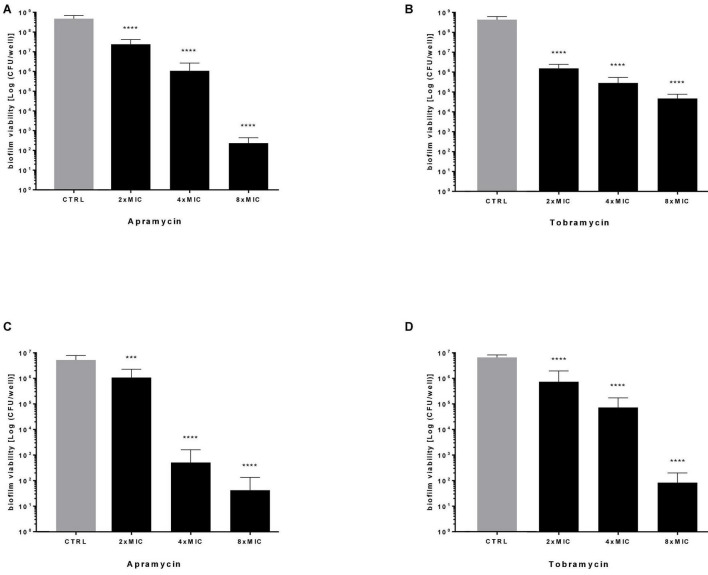
Killing activity against preformed *P. aeruginosa* biofilm. The efficacy of apramycin and tobramycin on the viability of 24 h-mature biofilm by *P. aeruginosa* Pa7 **(A,B)** and PaPh32 **(C,D)** was assessed by viable cell count assay. Each drug was tested at multiples of MIC value. Results are expressed as mean + SD of the residual biofilm viability [Log (CFU/well)] after 24 h-exposure. Control samples (CTRL) were not exposed to drug. Statistical significance at ordinary one-way ANOVA followed by Holm-Sidak’s multiple comparisons post-test: ***p < 0.001, ****p < 0.0001 vs. CTRL.

Next, the effect of antibiotics at multiples of MIC on the viability of preformed biofilm was evaluated by viable cell count assay, and results are summarized in Figure 5. Both drugs caused a highly significant, dose-dependent reduction of biofilm viability, regardless of strain tested. Particularly, when tested at 4x and 8xMIC against Pa7 mature biofilm, apramycin and tobramycin showed a killing rate > 99.9% (Figures 5A,B). Considered as a whole, our findings indicate that both antibiotics are effective in significantly reducing the viability of preformed biofilm, whereas stimulate EPS production in a strain-dependent manner.

**FIGURE 6:**
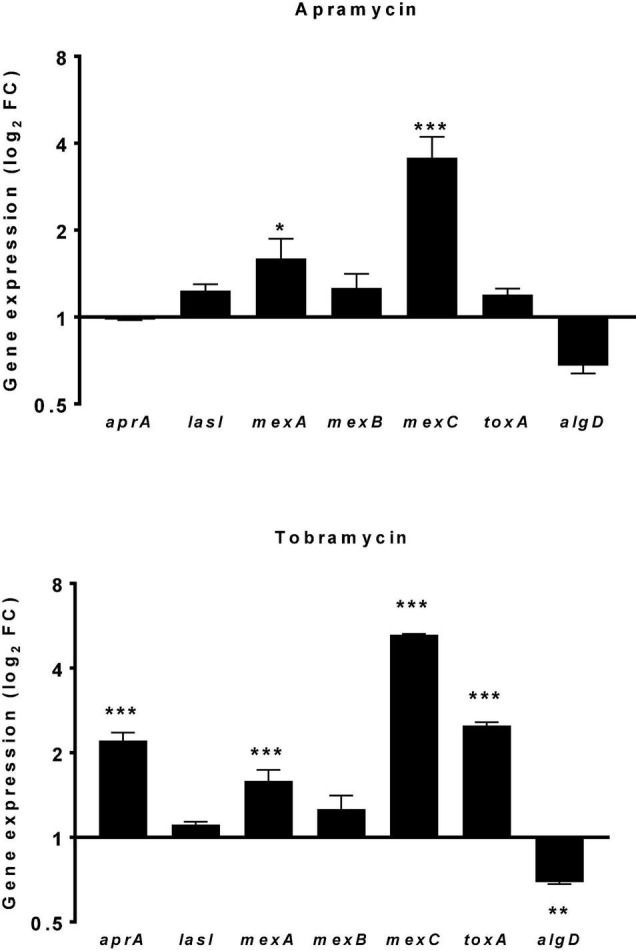
Effect of drug exposure on the expression of *P. aeruginosa* virulence genes. The effect of 20 h-exposure of apramycin and tobramycin at 0.25xMIC on the expression of *aprA* (alkaline protease), *lasI* (quorum sensing mediator), *mexA-B-C* (multidrug efflux pumps), *toxA* (exotoxin A), and *algD* (alginate synthesis) by *P. aeruginosa* PaPh32 was measured using the real-time RT-qPCR technique. Control samples (CTRL) were not exposed to drug. The relative expression of each gene was normalized to that of the housekeeping gene (*proC*). Results are presented as fold change (FC: 2^–ΔΔ*ct*^, mean ± SD) on a log_2_ scale. Statistical significance at ordinary one-way ANOVA followed by Tukey’s multiple comparisons post-test: *p < 0.05, **p < 0.01, and ***p < 0.001 vs. CTRL.

The rise in antibiotic resistance made urgently necessary the exploitation of alternative antibacterial strategies, such as antivirulence therapy (Kang et al., 2021). This approach might be useful for *P. aeruginosa* whose extensive repertoire of virulence factors, along with its adaptability, facilitate its persistence into the hostile environment of the CF airways (Jurado-Martín et al., 2021; Rossi et al., 2021). In this frame, the effect of apramycin on the expression of selected *P. aeruginosa* virulence genes (*aprA*, *lasI*, *mexA*, *mexB*, *mexC*, *toxA*, and *algD*) was evaluated comparatively to tobramycin, and results are resumed in Figure 6. A hallmark of *P. aeruginosa* isolates causing CF chronic infections is the multidrug resistance, mainly due to the presence of multidrug efflux (Mex) pumps (Lister et al., 2009; Llanes et al., 2011). Here we observed that both apramycin and tobramycin increase the expression of *mexA* (fold-change, mean ± SD: 1.596 ± 0.271 for apramycin, *p* < 0.05 vs. CTRL; 1.589 ± 0.148 for tobramycin, *p* < 0.001 vs. CTRL), and *mexC* (fold-change: 3.569 ± 0.647 for apramycin, *p* < 0.001 vs. CTRL; 5.258 ± 0.038 for tobramycin, *p* < 0.001 vs. CTRL), theoretically conferring cross-resistance to a broad range of antibiotics, such as beta-lactams, chloramphenicol, tetracycline, macrolides, novobiocin, fluoroquinolones, sulfamethoxazole, and trimethoprim (Masuda et al., 2000; Morita et al., 2001).

Of note, we observed that exposure to tobramycin might specifically raise the virulence potential in *P. aeruginosa* as suggested by the increased expression of *aprA* (fold-change: 2.211 ± 0.146, *p* < 0.001 vs. CTRL) and *toxA* (fold-change: 2.500 ± 0.071, *p* < 0.001 vs. CTRL), respectively, encoding for alkaline protease and exotoxin A. Alkaline protease is a zinc-dependent metallo-endopeptidase involved in phagocytic evasion, reducing mucocilliary bacterial clearance, and preventing flagellin-mediated immune recognition (Jurado-Martín et al., 2021). Exotoxin A, an ADP-ribosyl transferase inactivating protein synthesis (Michalska and Wolf, 2015), plays a role in establishing and the persistence of *P. aeruginosa* infection (Gallant et al., 2000). In CF patients it also stimulates epithelial cells and alveolar macrophages to produce group IIA secreted phospholipase A2 that selectively kills *S. aureus*, thus allowing P. *aeruginosa* to be progressively predominant in adult patients’ airways (Belmadi et al., 2018).

The exposure to apramycin and tobramycin specifically caused *algD* down expression in *P. aeruginosa*, although we observed statistical significance only in the case of tobramycin (fold-change: −1.441 ± 0.023, *p* < 0.01 vs. CTRL). This finding might be of relevance in the management of CF patients where the transition from acute to chronic lung infection is driven by the emergence of isolates showing a mucoid phenotype due to the overproduction of alginate codified by the operon *algD* (Bianconi et al., 2015). Indeed, alginate-overproducer *P. aeruginosa* strains promote the biofilm lifestyle and are therefore associated with poor prognosis and increased mortality (Smith et al., 2013; Malhotra et al., 2019).

The expression of other genes tested, *lasI* and *mexB*, were never significantly affected, regardless of antibiotic considered.

The use of aminoglycosides has been severely curtailed by important side effects, mainly ototoxicity and nephrotoxicity (Forge and Schacht, 2000). Recent *in-vitro* and *in-vivo* studies have indicated lower ototoxicity of apramycin, with absence of hidden hearing loss (Matt et al., 2012; Ishikawa et al., 2019), due to its exquisite selectivity for the bacterial over the eukaryotic cytosolic and mitochondrial ribosomes (Matt et al., 2012).

**FIGURE 7:**
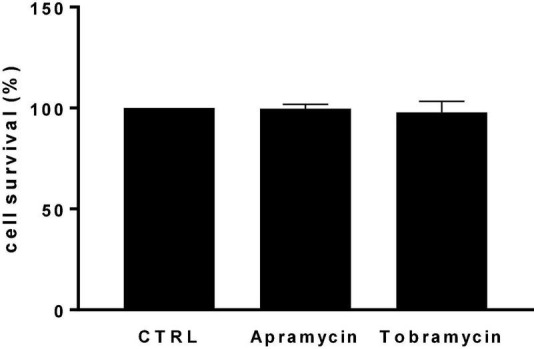
*In vitro* cytotoxicity against IB3-1 cells. IB3-1 cell monolayers were exposed for 24 h to apramycin and tobramycin at 512 mg/L. Control cells were not treated. At the end of incubation, the cell viability was assessed using an MTS tetrazolium-based colorimetric assay. Results are shown as mean + SD of the survival rate vs. control cells (CTRL, 100% cell survival). No statistically significant difference was found at Fisher’s exact test.

In this frame, evaluating the potential of apramycin for the treatment of CF lung infections requires assessing its cytotoxic potential toward the airway’s epithelium. For the first time in literature, the toxic potential of apramycin toward IB3-1 bronchial epithelial CF cells was assessed using a cell-based MTS assay. The exposure to apramycin and tobramycin at 512 mg/L—the highest concentration causing significant bactericidal and antibiofilm effects—did not cause any damage toward IB3-1 cells, whose growth was comparable to untreated control cells (Figure 7).

Despite the preliminary nature of our findings, we believe that apramycin may be worthy of consideration for repurposing in CF patients. Indeed, the rapid bactericidal activity and the low risk for acquired resistance we observed, along with the putative absence of typical aminoglycoside-associated ototoxicity reported earlier, point out the potential of apramycin, either directly and/or after derivatization, for development as an alternative treatment of *P. aeruginosa* infections. The efficacy against mature biofilms and the lack of toxicity toward CF bronchial cells are shared by apramycin and tobramycin. However, the higher activity exhibited by apramycin against mucoid strains along with the increased *P. aeruginosa* virulence following tobramycin exposure might make apramycin preferable in CF patients. Further human preclinical pharmacokinetic and pharmacodynamic studies are warranted to assess the clinical potential of our findings.

## Data Availability Statement

The original contributions presented in the study are included in the article/Supplementary Material, further inquiries can be directed to the corresponding author/s.

## Author Contributions

GD and AP: substantial contributions to the conception or design of the work, the acquisition, analysis, and interpretation of data for the work. GD, AP, FV, and GG: drafting the work and revising it critically for important intellectual content, provide approval for publication of the content. SG, VL, AP, FV, and RB: performing experimental activity. All authors contributed to the article and approved the submitted version.

## Conflict of Interest

The authors declare that the research was conducted in the absence of any commercial or financial relationships that could be construed as a potential conflict of interest.

## Publisher’s Note

All claims expressed in this article are solely those of the authors and do not necessarily represent those of their affiliated organizations, or those of the publisher, the editors and the reviewers. Any product that may be evaluated in this article, or claim that may be made by its manufacturer, is not guaranteed or endorsed by the publisher.
