# Supplementary material for: Repurposing the Veterinary Antibiotic Apramycin for Antibacterial and Antibiofilm Activity Against Pseudomonas aeruginosa From Cystic Fibrosis Patients
Source: Front Microbiol. 2022 Feb 3;12:801152. doi: 10.3389/fmicb.2021.801152 (PMC8851335; doi:10.3389/fmicb.2021.801152)
Supplement: Supplementary file 1 [file Table_1.pdf]

Table S1. **Primer sequences used in real-time RT-qPCR analyses**. The expression of selected virulence genes by *P. aeruginosa* PaPh32 was evaluated after 20 h-exposure to apramycin and tobramycin at 0.25xMIC. Primers were designed using as a reference the genome of *P. aeruginosa* strain NDTH9845 (GeneBank accession number: CP073080.1). All amplifications were performed using Ta = 60°C, and the gene *proC* was tested as housekeeping.

**Target gene**	**Primer sequences**	**RT-qPCR product (bp)**	**Gene function**
algD	F: 5'-CGACCTGGACCTGGGCTAC-3'<br>R: 5'-TCCTCGATCAGCGGGATC-3'	144	Alginate
toxA	F: 5'-TGGAGCGCAACTATCCCAC-3'<br>R: 5'-TAGCCGACGAACACATAGCC-3'	148	Exotoxin A
lasI	F: 5'-GAGCTTCTGCACGGCAAGG-3'<br>R: 5'-TTGATGGCGAAACGGCTGAG-3'	68	Quorum sensing
aprA	F: 5'-TACCTGATCAACAGCAGCTACAG-3'<br>R: 5'-GTAGCTCATCACCGAATAGGCG-3'	195	Alkaline protease
mexA	F: 5'-AGCAAGCAGCAGTACGCC-3'<br>R: 5'-GTGTAGCGCAGGTTGATCC-3'	86	Efflux pump
mexB	F: 5'-GCCTCGATCCATGAGGTAGTG-3'<br>R: 5'-AGGAACAGGTACATCACCAGG-3'	74	Efflux pump
mexC	F: 5'-ACGTCGGCGAACTGCAAC-3'<br>R: 5'-CTGAAGAAAGGCACCTTGGC-3'	101	Efflux pump
proC	F: 5'-AGGCCGGGCAGTTGCTGTC-3'<br>R: 5'-GTCAGGCGCGAGGCTGTC-3'	178	Proline biosynthesis
